# Silencing CACYBP suppresses lung adenocarcinoma growth via CDK1 inhibition

**DOI:** 10.17305/bb.2025.11849

**Published:** 2025-03-19

**Authors:** Ge Wen, Shaoqing Niu, Shiqi Mei, Senming Wang

**Affiliations:** 1 Department of Oncology, Zhujiang Hospital, Southern Medical University, Guangzhou, China; 2 Department of Radiation Oncology; Guangdong Provincial Key Laboratory of Major Obstetric Diseases; Guangdong Provincial Clinical Research Center for Obstetrics and Gynecology; The Third Affiliated Hospital, Guangzhou Medical University, Guangzhou, China; 3 Department of Radiation Oncology, First Affiliated Hospital of Sun Yat-sen University, Guangzhou, China; 4 Department of Oncology, The Second School of Clinical Medicine, Southern Medical University, Guangzhou, China

**Keywords:** Calcyclin-binding protein, CACYBP, lung adenocarcinoma, LUAD, cyclin-dependent kinase 1, CDK1, PI3K/AKT pathway, proliferation

## Abstract

Calcyclin-binding protein (CACYBP) is a multidomain adaptor protein implicated in the development of various cancers. However, its molecular and biological roles in lung adenocarcinoma (LUAD) remain unclear. In this study, we aimed to elucidate the biological impact of CACYBP in LUAD. Immunohistochemistry was used to assess CACYBP expression in LUAD tissues. Lentivirus-mediated CACYBP knockdown was established in LUAD cell lines, and target gene expression was analyzed via Western blotting and qRT-PCR. Cell proliferation, apoptosis, and migration were evaluated using flow cytometry, colony formation assays, cell counting kit-8 (CCK 8) assays, Celigo cell counting, wound healing assays, Transwell assays, and mouse xenograft models. Co-immunoprecipitation was performed to verify the interaction between CACYBP and cyclin-dependent kinase 1 (CDK1). Additionally, the phosphoinositide 3-kinase (PI3K) inhibitor LY294002 was used to investigate the involvement of CDK1 in the PI3K/AKT pathway. Our findings revealed that CACYBP was upregulated in LUAD tissues and correlated with advanced disease stages and poor prognosis. CACYBP knockdown inhibited LUAD progression and metastasis, promoted cell apoptosis *in vitro*, and reduced tumorigenicity *in vivo*. Mechanistically, we identified CDK1 as a direct interacting partner of CACYBP. CDK1 overexpression enhanced the malignant phenotype of LUAD cells and partially reversed the inhibitory effects of CACYBP knockdown. Furthermore, inhibition of the PI3K/AKT pathway using LY294002 significantly suppressed CDK1-mediated LUAD cell growth. In conclusion, CACYBP appears to function as a tumor promoter in LUAD, at least in part through CDK1-mediated activation of the PI3K/AKT pathway. These findings suggest that CACYBP could serve as a promising therapeutic target and a novel biomarker for LUAD prognosis.

## Introduction

Lung cancer has the highest incidence and mortality rates among all cancers globally, with non-small cell lung cancer (NSCLC) accounting for 80% to 85% of cases worldwide [1, 2]. Among the histological subtypes of NSCLC, lung adenocarcinoma (LUAD) is the most prevalent [3]. LUAD is typically asymptomatic in its early stages and is prone to metastasis and recurrence. As a result, most patients are diagnosed at an advanced stage, contributing to the high rate of cancer-related deaths [4, 5]. Although the advent of targeted therapies and immunotherapies—especially when combined with standard treatments—has improved clinical outcomes for LUAD patients, prognosis remains poor for many. This is largely due to low response rates to immunotherapeutic agents in some patients and the inevitable development of resistance to targeted drugs [6–8]. Therefore, elucidating the mechanisms that regulate LUAD development and progression is critical for identifying promising therapeutic targets and improving patient prognosis.

Calcyclin-binding protein (CACYBP) is a multidomain protein that interacts with various partners—including members of the S100 family, Siah-1, Skp1, tubulin, ERK1/2, and Nrdp1—via distinct binding sites. Through these interactions, CACYBP is involved in multiple cellular processes, such as protein ubiquitination, cytoskeletal rearrangement, cell proliferation, differentiation, autophagy, and cell cycle regulation [9–12]. Previous studies have demonstrated that CACYBP plays diverse roles in tumor development. For instance, it may act as a tumor promoter in cholangiocarcinoma, liver cancer, and prostate cancer [13–15]. Conversely, CACYBP appears to function as a negative regulator of cell proliferation in astrocytoma and renal cell carcinoma [16, 17], and may serve as a tumor suppressor in breast cancer [18]. However, another study has reported that CACYBP promotes breast carcinogenesis [19]. These findings suggest that while CACYBP plays a regulatory role in tumor growth and progression, its expression and function may vary significantly across different tumor types.

Recently, key genes associated with sphingomyelin metabolism, including CACYBP, have been identified through the integration of single-cell RNA-seq and bulk RNA-seq data, leading to the development of a prognostic prediction model for LUAD [20]. However, the precise function and underlying molecular mechanisms of CACYBP in LUAD remain unclear. Therefore, in this study, we investigated the biological role of CACYBP in LUAD cells by performing knockdown experiments and exploring the associated regulatory mechanisms.

## Materials and methods

### TCGA database analysis

The RNA-seq raw counts and clinical data for LUAD were obtained from The Cancer Genome Atlas (TCGA) via the Genomic Data Commons (GDC) data portal (https://portal.gdc.cancer.gov/) using the GDC-client. The data were then preprocessed using the R/Bioconductor package TCGAbiolinks [21]. In total, 524 tumor tissue samples and 59 normal tissue samples were available, including 57 paired samples. Subsequently, the expression data were normalized, and differentially expressed genes (DEGs) between LUAD and normal tissues were identified using the R/Bioconductor package DESeq2. DEGs were defined using the following thresholds: absolute fold change ≥ 1.5, *P* < 0.05, and false discovery rate (FDR) <0.05 [22].

### Immunohistochemistry (IHC) of clinical specimens

Paraffin-embedded specimens from 96 LUAD tissues and 87 adjacent noncancerous tissues were obtained, along with clinicopathologic information, from The Third Affiliated Hospital of Guangzhou Medical University. The study design was approved by the hospital’s Ethics Committee. After deparaffinization with xylene and rehydration through a graded alcohol series, the tissue sections were boiled in citrate buffer using an autoclave. The sections were then sequentially blocked with 3% H_2_O_2_ and goat serum, followed by overnight incubation at 4 ^∘^C with primary antibodies: anti-CACYBP (1:100, ab171972, Abcam), anti-Cyclin-dependent kinase 1 (CDK1) (1:100, HPA003387, Sigma), or anti-Ki-67 (1:200, ab16667, Abcam). After washing, the sections were incubated sequentially with an HRP-conjugated goat anti-rabbit secondary antibody (1:400, ab6721, Abcam) and DAB, followed by hematoxylin counterstaining. Images were then captured using an inverted microscope and evaluated based on staining intensity and percentage [23]. The proportion score was multiplied by the intensity score to obtain an immunoreactivity score ranging from 0 to 12. A score of 0–4 was considered low CACYBP expression, while a score of 5–12 indicated high CACYBP expression.

### Cell lines and culture conditions

For downstream analysis, the BEAS-2B cell line—normal human bronchial epithelial cells—was obtained from Cobioer (Nanjing, China). Human LUAD cell lines A549 and NCI-H1299, along with the lung squamous cell carcinoma cell line EBC-1, were obtained from the China Center for Type Culture Collection. Cells were cultured in the following media (all from Gibco; Thermo Fisher Scientific, Inc., Waltham, MA, USA): BEAS-2B in DMEM, A549 in Ham’s F-12K, NCI-H1299 in RPMI-1640, and EBC-1 in MEM. All media were supplemented with 10% fetal bovine serum (FBS). Cell cultures were maintained in a humidified incubator at 37 ^∘^C with 5% CO_2_.

### Plasmid construction and lentivirus preparation

Shanghai Bioscienceres Co., Ltd. (Shanghai, China) assisted in constructing all lentiviral vectors and helper plasmids. Interfering lentiviral vectors were generated by inserting three short hairpin RNA (shRNA) sequences targeting CACYBP into the BR-V108 plasmid.

The shCACYBP target sequences were shCACYBP-1, 5′–AGCCAAAGGAGACACGGAATT–3′; shCACYBP-2, 5′–ATGATATGAAGCGAACCATTA–3′; and shCACYBP-3, 5′–GAATCTAAATGGGAAGAGTTA–3′. In addition, we generated CDK1-overexpressing cells by cloning the CDK1 coding sequence into the LV013 plasmid. Cells transfected with the corresponding empty plasmid served as negative controls (shCtrl). Successful transfection was confirmed by the detection of green fluorescence under a fluorescence microscope.

### Quantitative reverse transcription polymerase chain reaction (qRT–PCR)

Total RNA was extracted from cultured cells using TRIzol Reagent (Sigma, Beijing, China). Reverse transcription into complementary DNA was performed using the HiScript Q RT SuperMix for qPCR (+gDNA wiper) Kit (Vazyme, Nanjing, China), following the manufacturer’s instructions. qRT-PCR was then carried out using the AceQ qPCR SYBR Green Master Mix Kit (Q111-02, Vazyme, Nanjing, China), according to the provided protocol. mRNA expression levels were quantified using the 2^−ΔΔCt^ method after normalization to the internal reference gene GAPDH. Primers used for qRT-PCR were synthesized by Sangon Biotech (Shanghai, China), and the corresponding sequences are listed in Table S1.

### Western blotting and co-immunoprecipitation (co-IP) assays

Total protein was extracted from cultured cells using RIPA lysis buffer (Beyotime Biotechnology, Shanghai, China). Protein concentrations were determined with the BCA Protein Assay Kit (Pierce; Thermo Fisher Scientific, Inc.). Equal amounts of protein (20 µg) were separated by 10% SDS-PAGE (Beyotime Biotechnology) and transferred to a PVDF membrane. Membranes were blocked with 5% skimmed milk in TBST at room temperature for 1 h, then incubated with the primary antibody overnight at 4 ^∘^C, followed by incubation with the secondary antibody for 2 h at room temperature. Immunoreactivity was detected using the enhanced chemiluminescence (ECL) method with the Immobilon Western Chemiluminescent HRP Substrate Kit (Millipore; Merck KGaA).

For co-IP, 1.0 mg of protein was first incubated with diluted antibodies at 4 ^∘^C overnight, followed by a 2-h incubation with 20 µL of magnetic beads. After centrifugation, the resulting pellet was washed with IP lysis buffer. The protein complexes were then denatured in IP lysis buffer and 5× loading buffer by boiling at 100 ^∘^C for 5 min. The resulting samples were analyzed by western blotting. Details of the antibodies used for western blotting and co-IP are provided in Table S2.

### Cell counting kit-8 (CCK-8) assay

A549 and NCI-H1299 cells were seeded into 96-well plates at a density of 2500 cells per well in 100 µL of medium, during their logarithmic growth phase, and subjected to various treatments or transfections. The cells were cultured at 37 ^∘^C in a humidified atmosphere containing 5% CO_2_. Starting from the second day after plating, 10 µL of CCK-8 reagent (Sigma; Merck KGaA) was added to each well prior to terminating the culture. After an additional 2-h incubation, the optical density of each well was measured at 450 nm using a Tecan Infinite 200 Pro microplate reader.

### Colony formation assay

Colony formation was assessed using lentivirus-transfected A549 and NCI-H1299 cells cultured in six-well plates (1000 cells/2 mL per well). Cells were incubated for eight days, with the medium refreshed every three days. After fixation with 4% paraformaldehyde and staining with Giemsa, colonies in the control and experimental groups were counted and analyzed.

### Celigo cell counting assay

A549 and NCI-H1299 cells were seeded into 96-well plates at a density of 2000 cells per well in 100 µL of medium. Only cells in the logarithmic growth phase that had undergone the relevant treatments or transfections were used. After seeding, the cells were incubated overnight at 37 ^∘^C in a 5% CO_2_ atmosphere. Cell numbers were monitored daily using a Celigo image cytometer (Nexcelom Bioscience, Lawrence, MA, USA), and proliferation curves were generated over five consecutive days, beginning on day two.

### Flow cytometry analysis of apoptosis

Apoptosis was assessed in lentivirus-transfected A549 and NCI-H1299 cells cultured in six-well plates (8 × 10^5^ cells/2 mL per well). After 96 h, the cells were harvested and stained using the Annexin V-allophycocyanin (APC) Apoptosis Detection Kit (Invitrogen; Thermo Fisher Scientific, Inc.), following the manufacturer’s protocol. Apoptotic cells were then analyzed via flow cytometry (Guava easyCyte HT, Millipore; Merck KGaA).

### Migration assays

Wound healing and Transwell assays were performed to assess the migratory ability of lentivirus-transfected A549 and NCI-H1299 cells.

For the wound healing assay, transfected cells (5 × 10^4^ cells/well) were seeded into 96-well plates and cultured until a confluent monolayer formed. Wounds were created using a 96-wounding replicator (V&P Scientific). After gently rinsing the monolayers three times with serum-free medium, 0.5% FBS was added to each well. Cell migration was then monitored by capturing images at 0 h and at subsequent time points. The distance between the migrating cell fronts was measured using ImageJ software.

We performed the assays using 24-well Transwell inserts (8-µm pore size, Corning). Transfected cells (5 × 10^4^ cells/well) were resuspended in 100 µL of serum-free medium and seeded into the upper chamber. The lower chamber was filled with 600 µL of medium containing 30% FBS. After 30 or 48 h of incubation, non-migrated cells were gently removed using a cotton swab. Migrated cells were fixed with 4% paraformaldehyde, stained with Giemsa, and imaged under an inverted microscope (IX73, Olympus). Cells were counted in five randomly selected fields.

### Mouse xenograft model

The animal experiments were approved by the Third Affiliated Hospital of Guangzhou Medical University (Approval No. S2021-120). All procedures complied with the Guidelines for the Ethical Review of Laboratory Animal Welfare, China (GB/T 35892-2018) [24]. A cohort of 20 four-week-old female BALB/c nude mice was obtained from Beijing Vital River Laboratory Animal Technology Co., Ltd. (Beijing, China). Before any experimental procedures, the mice were given time to acclimate to the laboratory environment. They were housed in groups of five per standard cage within an individually ventilated cage system, under controlled conditions: temperature (22 ^∘^C–24 ^∘^C), humidity (50%–60%), and a 12-hour light/dark cycle. High-quality corncob bedding was provided and replaced biweekly. The mice had ad libitum access to standard laboratory rodent chow and sterilized water, which was changed weekly. All procedures were performed under anesthesia, with strict measures taken to minimize distress and suffering.

Mice were randomly divided into two groups, shCACYBP and shCtrl, with 10 mice in each group. To establish mouse xenograft models, transfected NCI-H1299 cells (4 × 10^6^ cells suspended in 200 µL of PBS) were subcutaneously injected into the right axilla of each mouse. Tumor diameter and body weight were measured weekly, and tumor volume was calculated using the formula π/6 × length × width^2^. Predefined humane endpoints were set at a tumor volume ≥2000 mm^3^ or a weight loss ≥20%; however, none of the mice reached these thresholds during the study. On day 35 post-injection, *in vivo* fluorescence imaging of the tumors was performed using the IVIS Spectrum *In Vivo* Imaging System (PerkinElmer, Waltham, MA, USA). Following imaging, the mice were euthanized, and the excised tumor tissues were weighed and stored at −80 ^∘^C for further analysis.

### RNA screening analysis

Gene expression profiling and RNA screening analysis of NCI-H1299 cells transfected with shCACYBP or shCtrl were performed by Shanghai Bioscienceres Co., Ltd. (Shanghai, China). Total RNA was extracted using the TRIzol method and quantified with a NanoDrop 2000 system (Thermo Fisher Scientific, Inc.). Gene expression profiling was conducted using the Human Clariom S Assay (Affymetrix, Thermo Fisher Scientific, Inc.) according to the manufacturer’s protocol. Cartridge arrays were scanned and analyzed using a GeneChip^®^ Scanner 3000 7G System (Affymetrix, Thermo Fisher Scientific, Inc.). DEGs between NCI-H1299-shCACYBP and NCI-H1299-shCtrl cells were identified using a threshold of absolute fold change ≥ 1.5 and FDR <0.05. Enriched functional annotation and pathway analyses were performed using Ingenuity Pathway Analysis (IPA, Ingenuity Systems, Qiagen Inc.), with absolute Z-scores > 2 considered statistically significant.

### Ethical statement

This study design was approved by the Research Ethics Committee of the Third Affiliated Hospital of Guangzhou Medical University (Approval No. S2021-120), in accordance with the Declaration of Helsinki and the institution’s guidelines.

### Statistical analysis

Each experiment was performed independently at least three times. Data are presented as the mean ± standard deviation (SD). Statistical analyses were conducted using SPSS 26.0 and GraphPad Prism 9.0. Depending on the context, Student’s *t*-test, chi-square test, and analysis of variance (ANOVA) were employed for comparisons. Survival analysis was carried out using the Kaplan–Meier method with the log-rank test. Statistical significance was defined as a two-tailed *P* < 0.05. RNA-seq analysis was performed using the R/Bioconductor package “DESeq2,” which applies a negative binomial generalized linear model to identify DEGs [22]. To account for multiple comparisons inherent in RNA-seq data, the *P* < 0.05 threshold was adjusted using FDR control. DEGs were considered significant if they met the criteria of an absolute fold change ≥1.5 and FDR <0.05, following the Benjamini–Hochberg procedure for multiple testing correction [25].

## Results

### CACYBP was up-regulated in LUAD and correlated with poor prognosis

**Figure 1.**
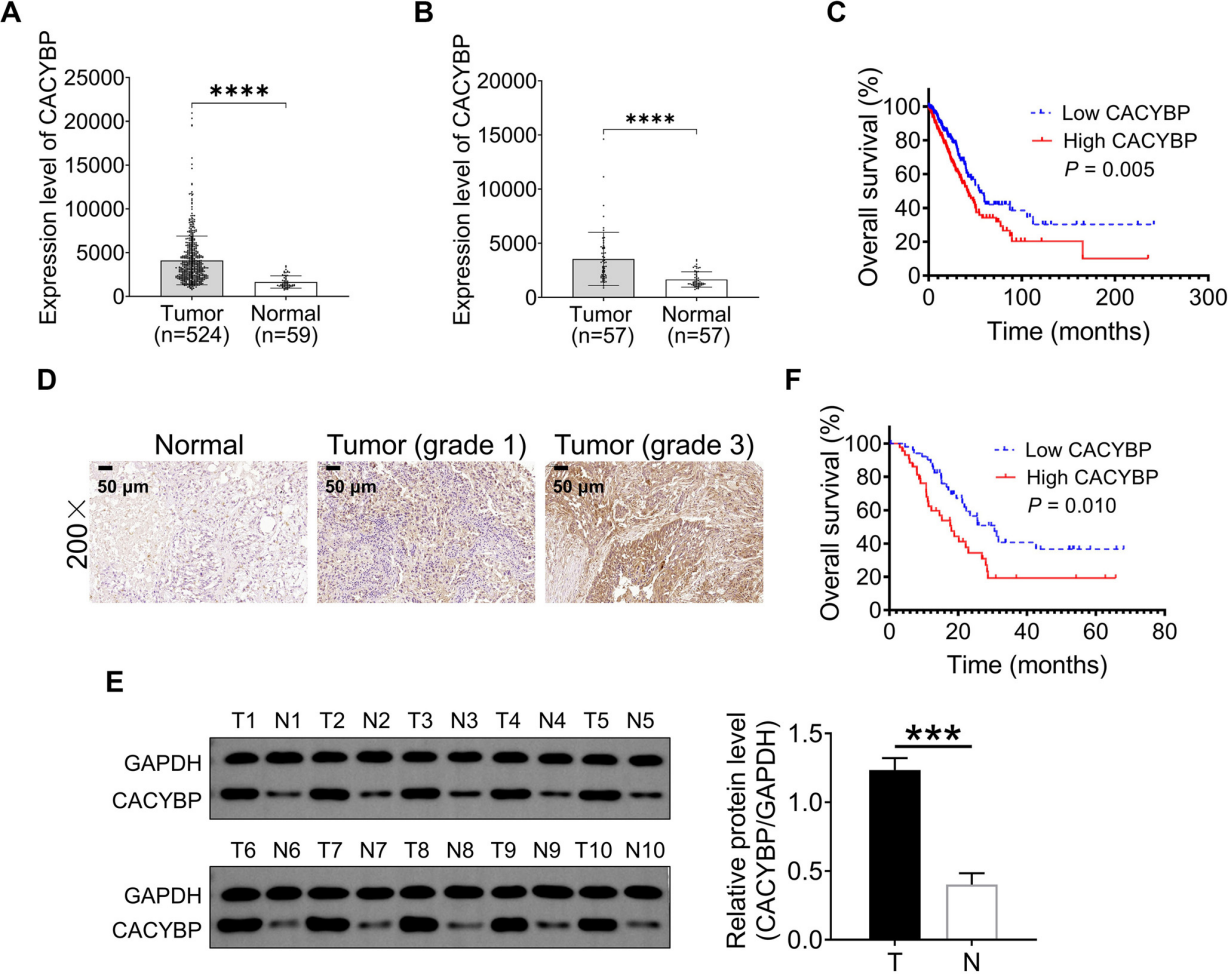
**CACYBP expression is upregulated in LUADand correlated with a poor prognosis.** (A and B) The distribution of CACYBP gene expression in LUAD tissue and normal tissue in the TCGA dataset, in which each point represents a sample, (A) population samples, (B) paired samples; (C) Overall survival curves predict a worse prognosis in LUAD patients with high CACYBP expression in the TCGA dataset; (D) The representative immunohistochemical CACYBP expression images of LUAD patients in adjacent normal and tumor tissues; (E) Western blotting was used to detect the expression of CACYBP in LUAD tumor tissues (T, *n* ═ 10) and adjacent normal tissues (N, *n* ═ 10); (F) Comparison of overall survival in LUAD patients with different levels of CACYBP expression. Data are shown as the mean ± SD and analyzed by paired Student’s *t*-test. ***P < 0.001 and ****P < 0.0001. CACYBP: Calcyclin-binding protein; LUAD: Lung adenocarcinoma; TCGA: The Cancer Genome Atlas; SD: Standard deviation.

Through analysis of RNA-seq count data from the TCGA-LUAD cohort, the CACYBP gene (ENSG00000116161) was found to be significantly upregulated in tumor samples compared to normal tissues (log_2_FC ═ 0.862, *P* ═ 3.99 × 10^−2^^4^). Gene expression levels of CACYBP in tumor vs normal tissues were assessed using a *t*-test. Results from both the overall cohort and a subset of 57 paired samples demonstrated that CACYBP mRNA expression was significantly higher in tumor tissues than in normal tissues (Figure 1A and 1B).

Among the 524 patients with LUAD, only 490 had complete clinical information, with a median age of 65 years (range: 33–88 years). These individuals were divided into low-expression and high-expression groups based on the median CACYBP gene expression levels in tumor samples. According to the chi-square test, the high-expression group had a significantly higher proportion of male patients. Additionally, CACYBP expression was associated with the TNM stage of LUAD patients (Table 1).

**Table 1:** Analysis of CACYBP expression and clinicopathological characteristics in LUAD patients from TCGA dataset

**Characteristics**	**No. of patients**	**CACYBP expression**		***P* value**
		**Low**	**High**	
All patients	490	244	246	
Age (years)				0.174
<62	236	110	126	
≥62	254	134	120	
Sex				0.014
Male	226	99	127	
Female	264	145	119	
TNM stage				0.033
I	265	141	124	
II	121	60	61	
III	81	38	43	
IV	23	5	18	

CACYBP: Calcyclin-binding protein; LUAD: Lung adenocarcinoma; TCGA: The Cancer Genome Atlas.

**Table 2:** Analysis of CACYBP expression and clinicopathological characteristics in LUAD patients

**Characteristics**	**No. of patients**	**CACYBP expression**		***P* value**
		**Low**	**High**	
All patients	96	52	44	
Age (years)				0.682
<62	48	27	21	
≥62	48	25	23	
Sex				0.409
Male	68	35	33	
Female	28	17	11	
Tumor size (cm)				0.079
<3.5	42	27	15	
≥3.5	54	25	29	
Grade				0.772
1	17	8	9	
2	55	30	25	
3	24	14	10	
TNM stage				0.041
I	35	25	10	
II	24	13	11	
III	31	12	19	
IV	6	2	4	

CACYBP: Calcyclin-binding protein; LUAD: Lung adenocarcinoma.

Furthermore, Kaplan–Meier survival analysis showed that patients with high CACYBP expression had significantly worse overall survival compared to those with low CACYBP expression (*P* ═ 0.005; Figure 1C). These findings suggest that CACYBP may play a role in the occurrence, progression, and prognosis of LUAD. To validate the bioinformatic predictions, Immunohistochemical tissue microarrays were used to assess CACYBP protein expression in 96 LUAD tissues and 87 paired adjacent noncancerous tissues. CACYBP expression was significantly higher in the cytoplasm of LUAD tissues (44/96, 45.8%) compared to normal tissues (8/87, 9.2%; *P* < 0.001) (Figure 1D). Additionally, CACYBP expression was upregulated in tumor tissues in ten paired LUAD samples (Figure 1E).

According to the IHC results, 96 LUAD patients with a median age of 62 years (range: 34–87 years) were divided into two groups based on CACYBP expression: a low-expression group (*n* ═ 52) and a high-expression group (*n* ═ 44). The relationship between CACYBP expression and the clinicopathological characteristics of LUAD patients was further analyzed. The results indicated that abnormal CACYBP expression was significantly associated with TNM stage in LUAD patients (Table 2).

As of September 18, 2023, follow-up information was available for 72 patients (75.0%). The median survival time was 19.5 months for the 39 patients in the low-expression group and 13.6 months for the 33 patients in the high-expression group. Kaplan–Meier survival analysis showed that high CACYBP expression was significantly associated with poorer overall survival in LUAD patients (*P* ═ 0.010; Figure 1F). These results further validated the bioinformatic predictions.

### Construction of CACYBP knockdown lung cancer cell lines

**Figure 2.**
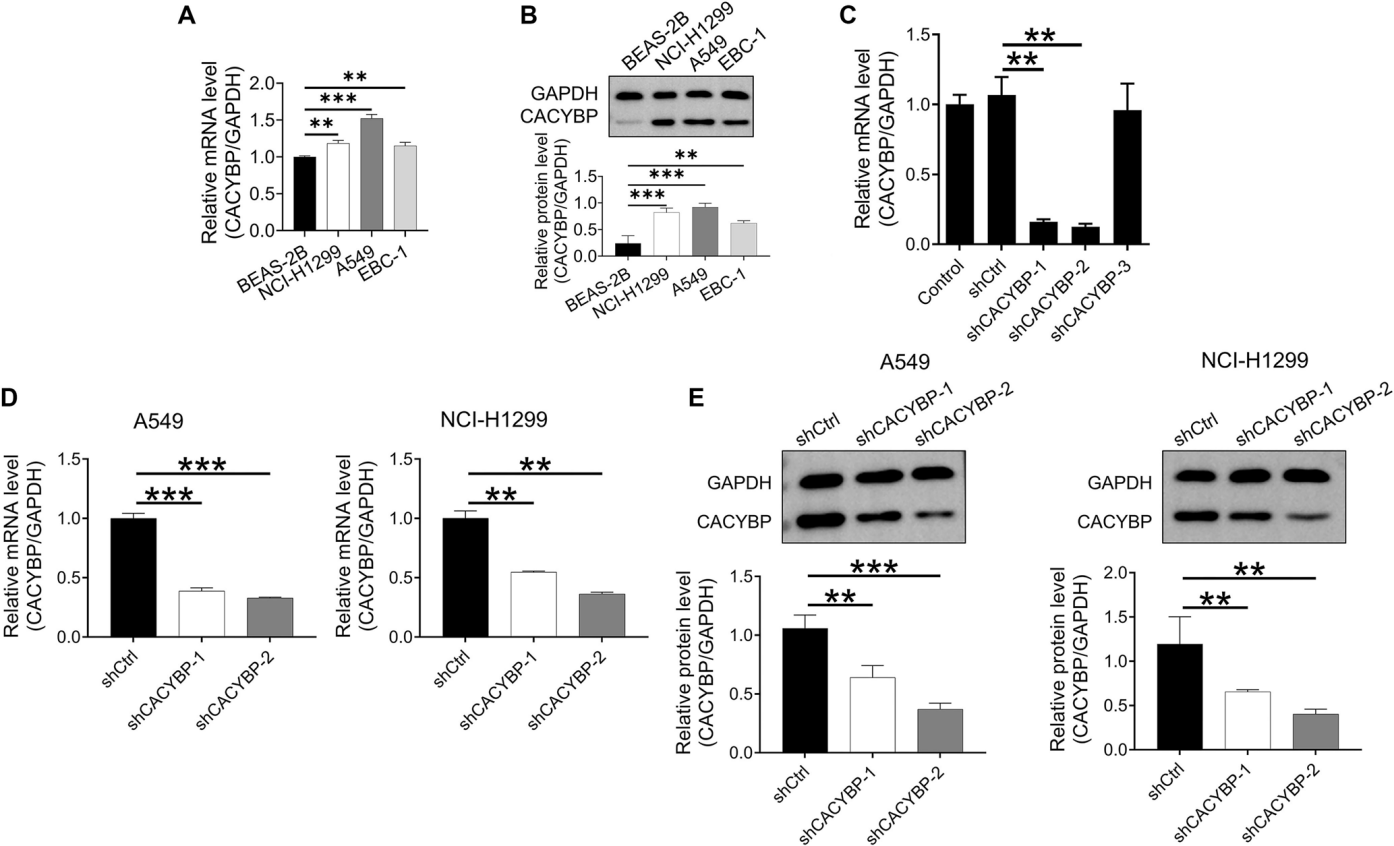
**Development of lung cancer cell lines with CACYBP knockdown.** (A and B) qRT-PCR and western blotting were used to detect the expression of CACYBP in the BEAS-2B, NCI-H1299, A549, and EBC-1 cell lines; (C) Using qRT–PCR, CACYBP knockdown effectiveness was assessed in NCI-H1299 cells; (D) qRT–PCR analysis of CACYBP expression in A549 and NCI-H1299 cell lines after transfection; (E) CACYBP protein expression in the A549 and NCI-H1299 cell lines was detected by western blotting after transfection. All data are shown as the mean ± SD (*n* ═ 3) and analyzed by unpaired Student’s *t*-test. **P < 0.01, ***P < 0.001. CACYBP: Calcyclin-binding protein; qRT–PCR: Quantitative reverse transcription polymerase chain reaction; SD: Standard deviation.

Given the correlation between CACYBP expression and both prognosis and clinicopathologic features, we sought to investigate its potential role in promoting malignant biological behavior in LUAD cell lines. CACYBP expression was found to be elevated in human NSCLC cell lines (NCI-H1299, A549, and EBC-1) compared to the normal bronchial epithelial cell line BEAS-2B (Figure 2A and 2B). To assess its function, three shRNAs targeting CACYBP were designed and synthesized for knockdown experiments in NCI-H1299 cells. qRT–PCR analysis revealed that CACYBP mRNA levels were reduced by 85.0% in the shCACYBP-1 group and by 88.3% in the shCACYBP-2 group, while the shCACYBP-3 group showed no significant knockdown (Figure 2C). To minimize off-target effects, two independent shRNAs (shCACYBP-1 and shCACYBP-2) were selected to establish stable cell lines with CACYBP suppression.

After 72 h of lentivirus-mediated transfection, shCACYBP-1 and shCACYBP-2 significantly reduced CACYBP mRNA and protein expression in A549 and NCI-H1299 cell lines (Figure 2D and 2E). Therefore, these cells were suitable for subsequent cellular function assays.

### CACYBP knockdown inhibits progression-related processes in LUAD cells *in vitro*

**Figure 3.**
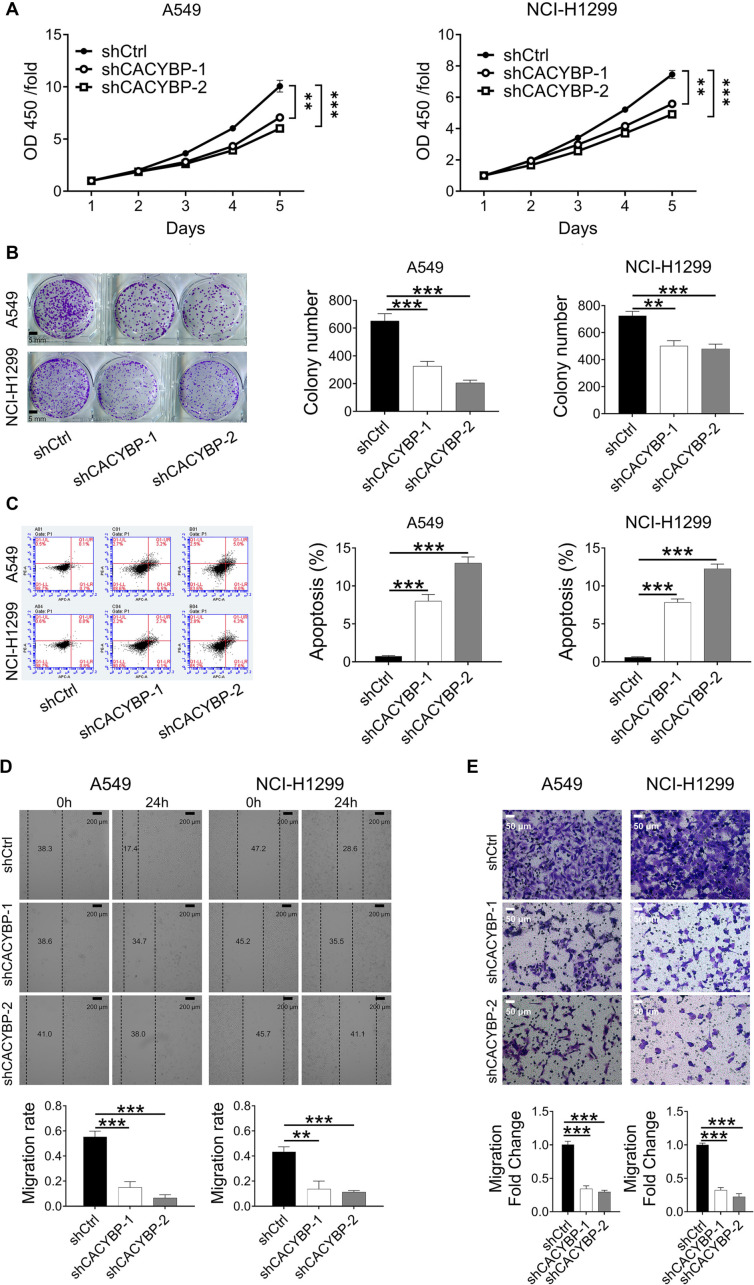
**CACYBP knockdown restricted LUAD cell progression *in vitro*.** (A and B) CCK-8 and colony formation assays indicated the effects of CACYBP knockdown on A549 and NCI-H1299 cell proliferation; (C) The effects of CACYBP knockdown on cell apoptosis were evaluated using flow cytometry; (D and E) The effects of CACYBP knockdown on the migratory capability of A549 and NCI-H1299 cells were assessed using wound healing assays (magnification: ×50) and Transwell assays (magnification: ×200). All data are shown as the mean ± SD (*n* ═ 3) and analyzed by unpaired Student’s *t*-test. **P < 0.01, ***P < 0.001. CACYBP: Calcyclin-binding protein; LUAD: Lung adenocarcinoma; CCK-8: Cell counting kit-8; SD: Standard deviation.

CCK-8 and colony formation assays demonstrated that silencing CACYBP significantly inhibited the proliferation of A549 and NCI-H1299 cells (Figure 3A and 3B). In addition, flow cytometry results indicated that CACYBP silencing induced apoptosis in both cell lines (Figure 3C). To assess the impact of CACYBP knockdown on cell migration, wound healing and Transwell assays were performed, revealing a marked reduction in A549 and NCI-H1299 cell migration (Figure 3D and 3E). Collectively, these *in vitro* experiments showed that CACYBP downregulation suppressed LUAD cell proliferation and migration while promoting apoptosis. Among the knockdown constructs, shCACYBP-2 was selected for further experiments due to its superior silencing efficiency.

### CACYBP knockdown inhibits LUAD tumor growth *in vivo*

**Figure 4.**
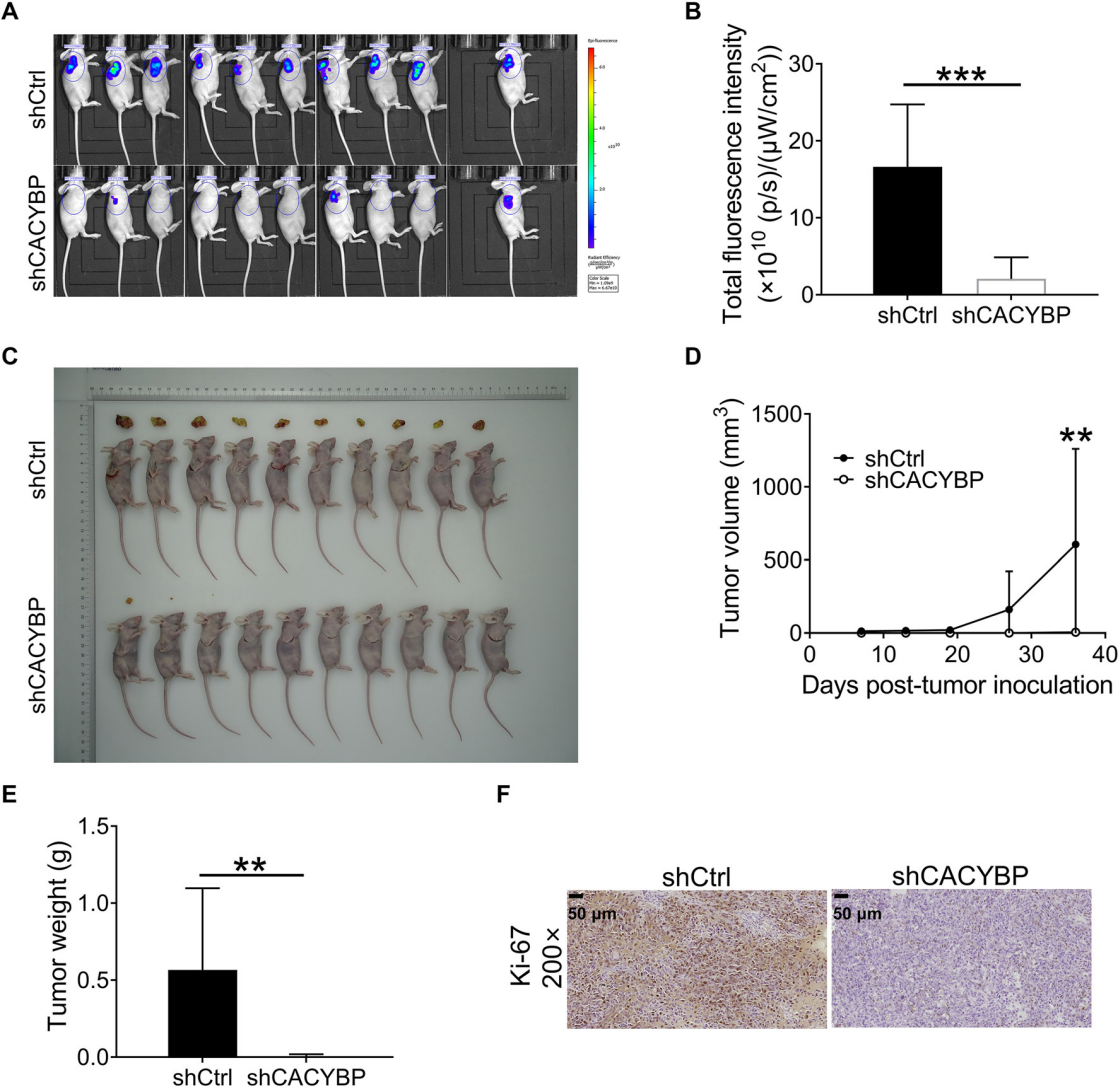
**CACYBP knockdown restricts NCI-H1299 cell growth *in vivo*.** (A) *In vivo* imaging was used to evaluate the tumor burden in the shCtrl and shCACYBP groups prior to euthanasia of mice; (B) Total fluorescence tumor intensity in both groups; (C) Photograph of resected xenograft tumors from mice in both groups; (D) Tumor volumes in both groups were determined during the experiment; (E) Tumor weight in both groups was measured after euthanasia of mice; (F) Evaluation of the Ki-67 index in resected tumors using IHC staining. **P < 0.01, ***P < 0.001. CACYBP: Calcyclin-binding protein; IHC: Immunohistochemistry.

The effect of CACYBP knockdown on LUAD proliferation was validated *in vivo* using mouse xenograft models established via subcutaneous injection of transfected NCI-H1299 cells with either shCACYBP-2 or shCtrl. Tumor fluorescence intensity was markedly reduced in the shCACYBP group compared to the shCtrl group (Figure 4A and 4B). Additionally, the shCACYBP group exhibited significantly fewer tumors, along with reduced tumor volume and weight, based on analyses of resected samples (Figure 4C–4E). Immunohistochemical staining further demonstrated decreased expression of the nuclear proliferation marker Ki-67 in the shCACYBP group (Figure 4F). Collectively, these findings indicate that CACYBP knockdown suppresses LUAD proliferation *in vivo*.

### CACYBP depletion inhibited LUAD progression via CDK1

**Figure 5.**
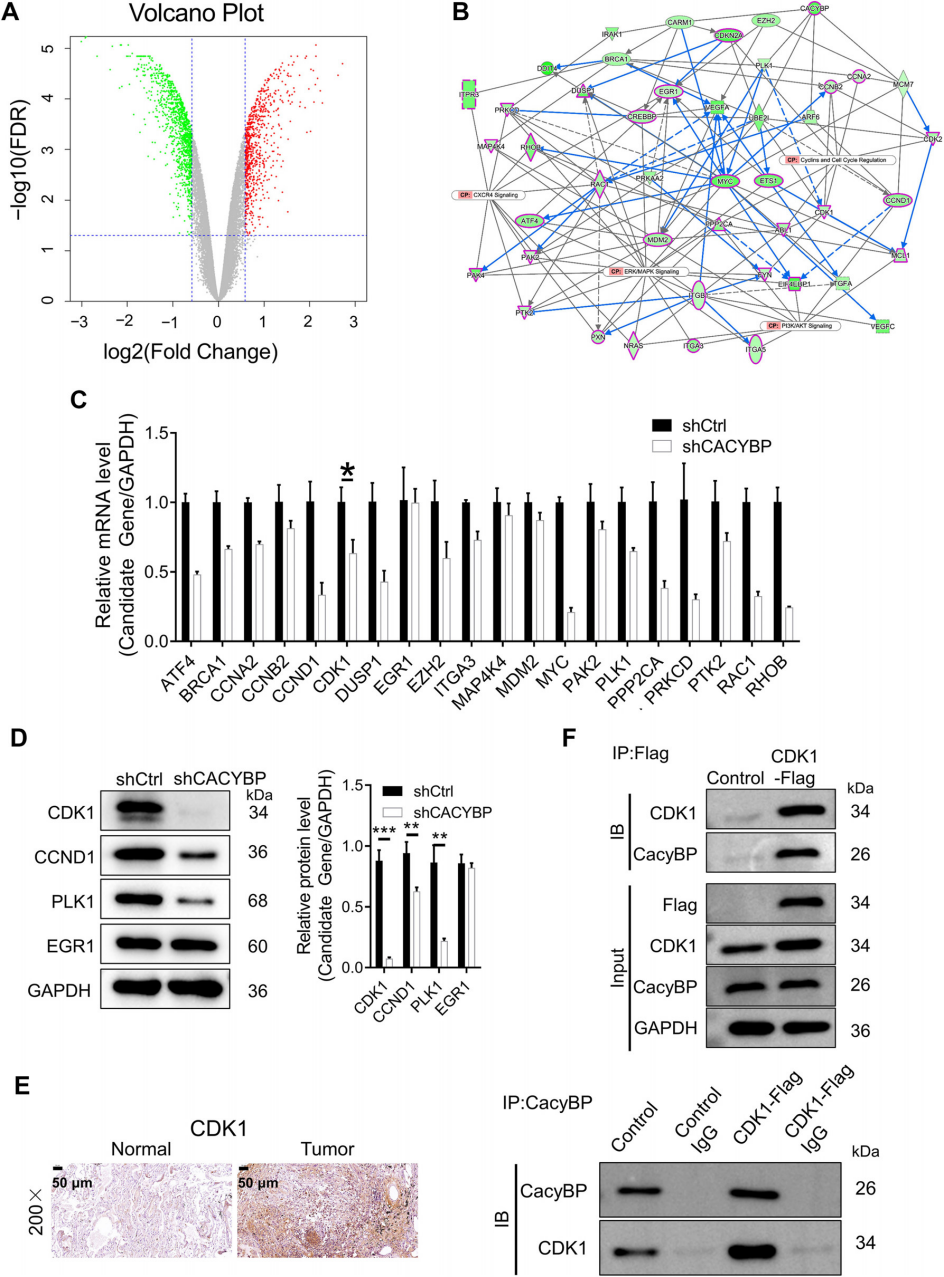
**Exploring the downstream CACYBP mechanism in NCI-H1299 cells.** (A) Volcano plot of DEGs between the experimental (shCACYBP) and control (shCtrl) groups in NCI-H1299 cells, red dots represent upregulated genes, green dots represent down-regulated genes, and gray dots represent genes with no significant difference; (B) Interaction network between CACYBP-associated DEGs established using IPA; (C and D) The expression of several candidate genes was examined using qRT–PCR and western blotting in NCI-H1299 cells transfected with or without shCACYBP, data are shown as the mean ± SD (*n* ═ 3) and analyzed by unpaired Student’s *t*-test; (E) CDK1 expression was determined in LUAD tissues and normal tissues using IHC staining; (F) A co-IP assay was used to validate the CDK1 and CACYBP interaction; Control: NCI-H1299 cells were transfected using an empty vector; CDK1-Flag: NCI-H1299 cells transfected with FLAG-tagged CDK1-overexpressing lentivirus. *P < 0.05, **P < 0.01, ***P < 0.001. FDR: False discovery rate; CACYBP: Calcyclin-binding protein; CDK1: Cyclin dependent kinase 1; DEGs: Differentially expressed genes; IPA: Ingenuity pathway analysis; LUAD: Lung adenocarcinoma; IHC: Immunohistochemistry; IP: Immunoprecipitation; SD: Standard deviation.

RNA-seq was performed to identify DEGs in NCI-H1299 cells transfected with either shCACYBP or shCtrl. A total of 1823 DEGs were identified: 731 were upregulated and 1092 were downregulated in shCACYBP cells compared to shCtrl cells (Figure 5A). Subsequently, IPA was used to assess DEG enrichment in canonical signaling pathways (Figure S1A) as well as in disease and functional categories (Figure S1B). Canonical pathway analysis revealed significant inhibition of several cancer-related pathways, including ERK/MAPK, phosphoinositide 3-kinase (PI3K)/AKT, and CXCR4 signaling (Figure S1A). The most prominent enrichment of DEGs was observed in the “Cancer” and “Organismal Injury and Abnormalities” categories (Figure S1B). Interaction network analysis based on the enrichment results of all DEGs indicated that CACYBP may indirectly regulate downstream genes involved in ERK/MAPK signaling, PI3K/AKT signaling, CXCR4 signaling, and cyclin and cell cycle regulation pathways (Figure 5B).

Based on the interaction network, 20 down-regulated downstream genes were selected for qRT-PCR verification. Additionally, Western blotting was used to confirm the results for four of these genes. The findings revealed that the shCACYBP group exhibited reduced mRNA and protein expression levels of CDK1, CCND1, and PLK1, while EGR1 levels remained unchanged (Figure 5C and 5D). Among these proteins, CDK1 is a key regulator of the cell cycle and was identified as a promising target candidate of CACYBP.

IHC staining and co-IP assays were conducted to validate this hypothesis. CDK1 protein expression was significantly higher in LUAD tissues compared to adjacent normal tissues (Figure 5E), and the co-IP assay confirmed a protein–protein interaction between CACYBP and CDK1 (Figure 5F). These findings suggest that CDK1 expression in LUAD follows a similar pattern to that of CACYBP. As a result, CDK1 was identified as a downstream target of CACYBP involved in the regulation of NCI-H1299 cells, and this relationship was further validated through *in vitro* experiments.

### CDK1 overexpression rescued CACYBP knockdown-mediated inhibition of LUAD

**Figure 6.**
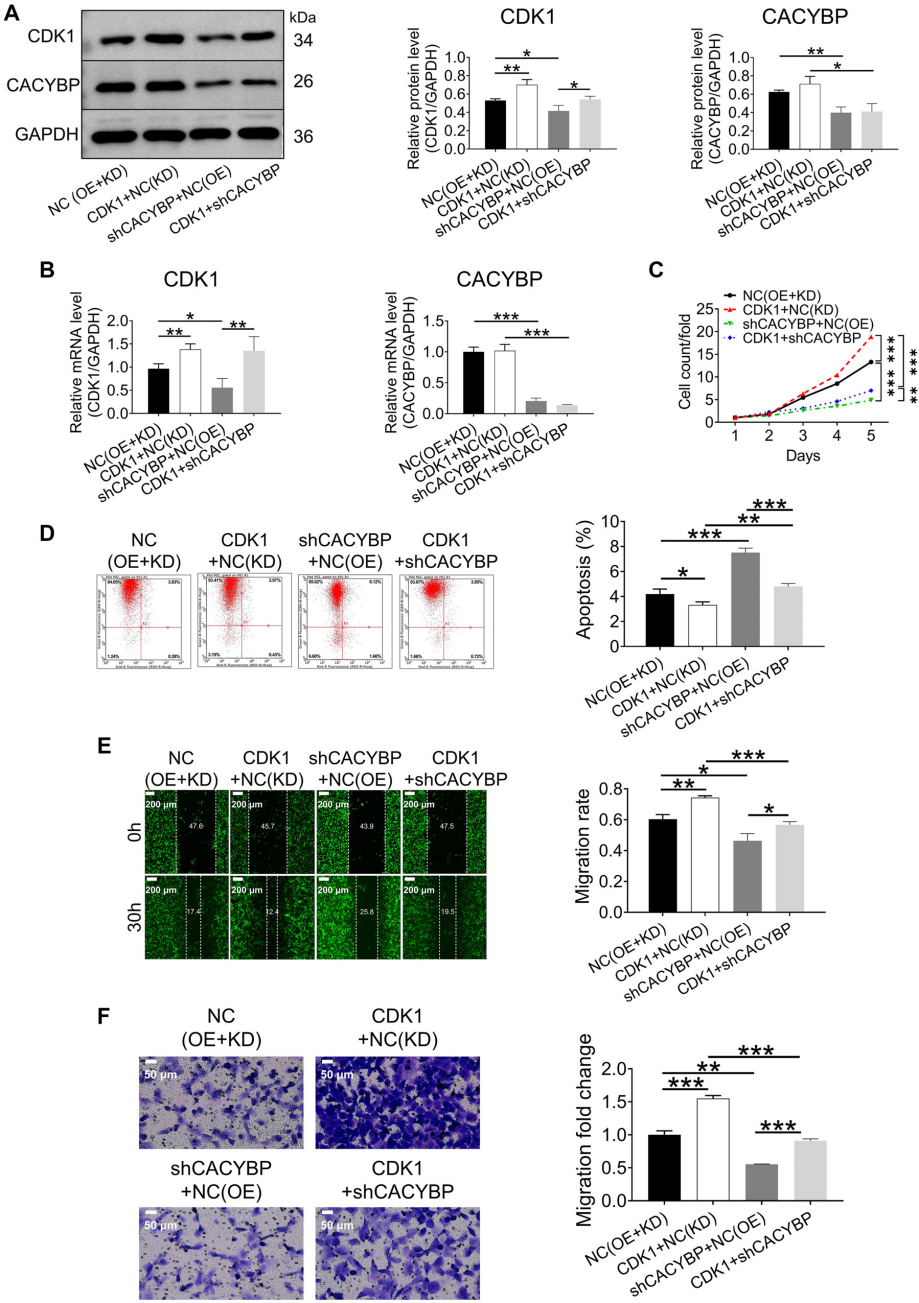
**CDK1 overexpression prevented the inhibitory effects of CACYBP knockdown on NCI-H1299 cells.** (A and B) Western blotting and qRT-PCR were used to detect the expression of CDK1 and CACYBP in NCI-H1299 cell models; (C) Cell proliferation was assessed using the Celigo cell counting assay; (D) Flow cytometry was used to determine cell apoptosis; (E and F) Wound healing (employing GFP as reporter gene, magnification: ×50) and Transwell (magnification: ×200) tests were used to measure the capacity of cells to migrate. All data are shown as the mean ± SD (*n* ═ 3) and analyzed by one-way ANOVA. *P < 0.05, **P < 0.01, ***P < 0.001. CACYBP: Calcyclin-binding protein; CDK1: Cyclin dependent kinase 1; GFP: Green fluorescent protein; ANOVA: Analysis of variance; SD: Standard deviation.

Rescue assays were conducted to evaluate the synergistic roles of CDK1 and CACYBP in LUAD progression. qRT–PCR analysis confirmed that CDK1 mRNA expression was significantly higher in NCI-H1299, A549, and EBC-1 cells compared to BEAS-2B cells (Figure S2A). Based on this, NCI-H1299 cell models were established to include the following groups: CDK1 overexpression only (CDK1+NC (KD)), CACYBP knockdown only (shCACYBP+NC (OE)), simultaneous CDK1 overexpression and CACYBP knockdown (CDK1+shCACYBP), and a negative control (NC (OE+KD)) (Figure 6A and 6B, and Figure S2B). Functional assays revealed that CDK1 overexpression promoted cell proliferation (Figure 6C and Figure S2C) and migration (Figure 6E and 6F), while inhibiting apoptosis (Figure 6D) in NCI-H1299 cells. Moreover, CDK1 upregulation partially rescued the inhibitory effects of CACYBP knockdown on proliferation and migration, as well as the increase in apoptosis (Figure 6C–6F and Figure S2C). These findings suggest that CACYBP may regulate LUAD progression, at least in part, through CDK1.

### CDK1 promotes the growth of LUAD cells in a PI3K/AKT pathway-dependent manner

**Figure 7.**
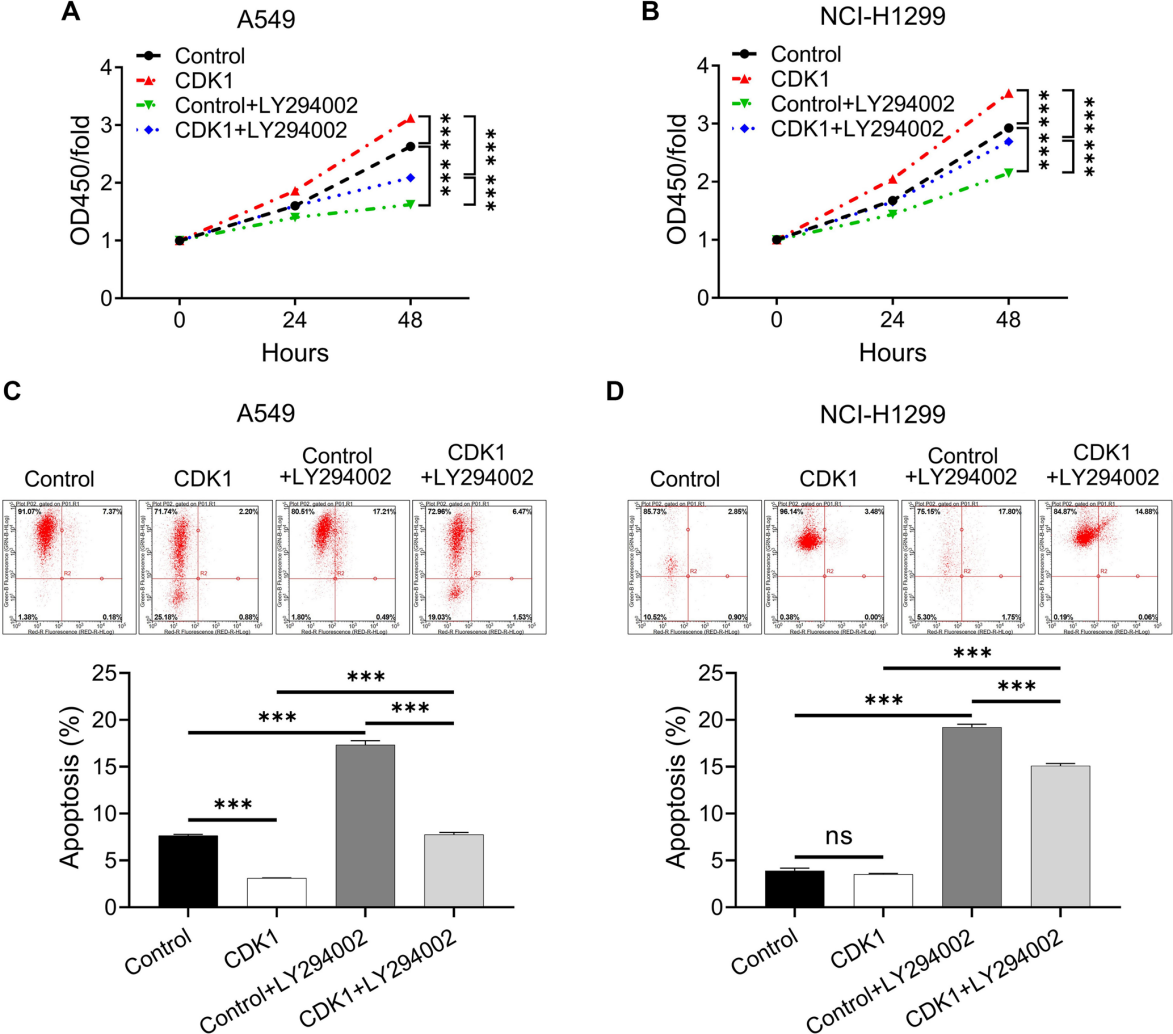
**The effects of CDK1 on LUAD cell growth were carried out via the PI3K/AKT pathway.** Following a 24-h treatment with LY294002 (50 µM), (A and B) Cell proliferation; (C and D) Cell apoptosis were measured using the CCK-8 assay and flow cytometry, respectively. All data are shown as the mean ± SD (*n* ═ 3) and analyzed by one-way ANOVA. ***P < 0.001. ns: No significance; OD: Optical density; CDK1: Cyclin dependent kinase 1; LUAD: Lung adenocarcinoma; PI3K: Phosphatidylinositol 3-kinase; AKT: Protein kinase B; CCK-8: Cell counting kit-8; ANOVA: Analysis of variance; SD: Standard deviation.

We investigated whether the effect of CDK1 on LUAD cells is mediated through the PI3K/AKT pathway, which was inhibited by shCACYBP according to IPA analysis. To test this, rescue experiments were conducted using the PI3K inhibitor LY294002 to evaluate proliferation and apoptosis in A549 and NCI-H1299 cells. Treatment with LY294002 attenuated CDK1-induced proliferation and reduced its inhibitory effect on apoptosis in both cell lines (Figure 7A–7D). These findings suggest that CDK1 regulates LUAD cell growth through the PI3K/AKT pathway.

## Discussion

CACYBP is differentially expressed across human tissues, with high expression observed in the brain, heart, and esophagus, and low or undetectable levels in most other normal tissues. However, it is aberrantly expressed in the majority of tumor tissues [26]. Chen et al. [27] reported that in pancreatic cancer, elevated CACYBP expression is significantly associated with poor differentiation, advanced TNM stage, and distant metastasis. Similarly, Lian et al. [15] found that high CACYBP expression in hepatocellular carcinoma was linked to significantly reduced overall and disease-free survival. Using the TCGA dataset, we observed that CACYBP mRNA expression was significantly upregulated in LUAD clinical samples compared to normal lung tissues. Moreover, its expression correlated with tumor stage, and patients with high CACYBP expression exhibited poorer survival outcomes. These findings suggest that CACYBP may be associated with the malignant phenotype of tumors.

In the present study, IHC was used to assess CACYBP expression in clinical samples from patients with LUAD. We found that CACYBP protein expression was significantly higher in LUAD tissues compared to adjacent noncancerous tissues. Positive CACYBP expression in LUAD was localized to the cytoplasm and was associated with advanced clinicopathological stage, but showed no correlation with age, sex, tumor size, or pathological grade. Additionally, high CACYBP expression was linked to poor prognosis in LUAD patients. These findings suggest that CACYBP may function as a tumor promoter in LUAD; however, its role in LUAD tumorigenesis and progression remains underreported and not fully understood.

CACYBP, a multi-ligand protein, exhibits tissue-specific expression profiles and context-dependent roles in tumorigenesis. A comprehensive pan-cancer analysis identified CACYBP as being upregulated in 14 cancers—including lung, liver, colon, pancreatic cancers, and cholangiocarcinoma—while it was downregulated in six cancers, such as kidney renal clear cell carcinoma and prostate cancer. This highlights its dual functionality as either an oncogene or a tumor suppressor, depending on the cancer type [28]. For example, in liver cancer, CACYBP promotes cell progression by modulating p27ˆKip1 and cyclins [15]. In contrast, its knockdown in prostate cancer impedes cancer progression by upregulating p53, suggesting an oncogenic function in this context [14]. Similarly, CACYBP knockdown in pancreatic cancer inhibits cell growth by blocking the G1-to-S phase transition, mediated by the downregulation of Cyclin E, Cyclin A, and CDK2, along with the upregulation of p27ˆKip1 and Rb [29]. In colon cancer, CACYBP enhances proliferation by interacting with Skp1 to degrade p27ˆKip1 [30], while in cholangiocarcinoma, it promotes progression by inhibiting MCM2 ubiquitination and activating the Wnt/β-catenin signaling pathway [13]. Conversely, in astrocytoma patients, higher CACYBP expression is associated with a favorable prognosis, indicating a tumor-suppressive function [16]. In renal cancer, CACYBP overexpression reduces Cyclin D1 levels through β-catenin degradation, thereby inhibiting cell growth and tumorigenicity—further supporting its tumor-suppressive role [17]. Notably, CACYBP’s role in gastric cancer, glioma, and breast cancer remains controversial. In gastric cancer, some studies suggest it promotes proliferation by binding Skp1, degrading p27ˆKip1, and increasing Cyclin E [31, 32]. However, other studies report it may inhibit gastric cancer cell proliferation by degrading β-catenin and dephosphorylating ERK1/2 [33, 34]. In glioma, CACYBP appears to have an oncogenic role by enhancing proliferation and reducing apoptosis via downregulation of p53 and p21, alongside activation of Akt, β-catenin, and ERK1/2 [35, 36]. Nevertheless, it may also suppress glioma cell migration and invasion through Siah1-mediated cytoplasmic downregulation of p27ˆKip1 [37]. In breast cancer, CACYBP knockdown has been shown to increase proliferation and invasion by upregulating COX-2 [18], though other studies suggest that CACYBP expression may actually facilitate breast cancer progression [38]. The observed discrepancies in CACYBP’s role across different cancers may be attributed to specific cellular contexts and the involvement of distinct molecular pathways [28].

In this study, we found that CACYBP knockdown significantly hindered LUAD cell progression both *in vitro* and *in vivo* by reducing proliferation, promoting apoptosis, and limiting migration and invasion. These findings support CACYBP’s role as a critical oncogenic driver in LUAD, consistent with its oncogenic function in other malignancies, such as liver, prostate, pancreatic, and colon cancers, as well as cholangiocarcinoma [13–15, 29, 30]. Our results also showed that high CACYBP expression correlates with advanced neoplasia and poor prognosis, suggesting its potential as a predictive biomarker for identifying high-risk LUAD patients who may benefit from more aggressive treatment. Similar biomarker-based stratification has proven effective in guiding immunotherapy decisions through PD-L1 expression in NSCLC [39]. Moreover, our data demonstrate that CACYBP knockdown suppresses tumor growth in both *in vitro* and *in vivo* models, highlighting its potential as a therapeutic target. Development of small-molecule inhibitors or RNA interference (RNAi)-based therapies—such as siRNA or shRNA delivered via lipid nanoparticles—could be pursued to silence CACYBP expression, thereby disrupting its protein interactions and downstream oncogenic effects [40, 41]. Future research should prioritize the development of selective and safe CACYBP inhibitors, leveraging artificial intelligence and machine learning in computational chemistry and molecular docking to identify novel candidates and facilitate their clinical translation.

Gene expression profiling was conducted to investigate the molecular mechanism by which CACYBP regulates LUAD using bioinformatic analysis, identifying CDK1 as a potential target. Subsequent validation showed that CDK1 expression was significantly elevated in LUAD tissues compared to normal tissues. Notably, co-IP assays in NCI-H1299 cells demonstrated an interaction between CACYBP and CDK1, indicating that CDK1 is a downstream target of CACYBP involved in regulating NCI-H1299 cells.

CDK1 is a member of the serine/threonine kinase family and serves as a pleiotropic regulator of the cell cycle. It drives cells through the G2 phase and into mitosis by interacting with its catalytic partner, cyclin B [42]. Beyond its role in cell cycle regulation, CDK1 also acts as a translational activator, enabling protein synthesis to adapt directly to the cell proliferation rate [43]. CDK1 may be associated with the malignant phenotype of lung cancer. For example, elevated CDK1 transcript and protein levels have been observed in lung cancer samples compared to normal tissue. Furthermore, its increased expression correlates with advanced tumor stage and poorer survival outcomes [44]. CDK1 also mediates the oncogenic effects of NUCKS1 overexpression, promoting proliferation, invasion, and migration in NSCLC cells [45]. This study demonstrated that CDK1 overexpression enhances LUAD cell growth and motility while inhibiting apoptosis. Additionally, CDK1 overexpression counteracts the suppressive effects of CACYBP knockdown on LUAD cell development, linking CDK1 to CACYBP-driven regulation in LUAD.

Our study demonstrates that CACYBP directly interacts with CDK1, as shown by co-IP assays. Targeting the CACYBP–CDK1 complex may represent a potential therapeutic strategy for LUAD. Although protein–protein interactions like CACYBP–CDK1 are typically challenging to target, high-throughput screening and structure-based drug design approaches could facilitate the discovery of small molecules capable of disrupting this interaction [46, 47]. Existing pan-CDK inhibitors, such as Dinaciclib and Seliciclib, which target multiple CDKs, may hold promise for LUAD, particularly in tumors that are highly dependent on CDK1 activity [48]. Dinaciclib has demonstrated efficacy in clinical trials for leukemia, breast, and pancreatic cancers [49–51], while Seliciclib has been evaluated in various cancer types [52, 53]. RO-3306, a selective CDK1 inhibitor, has shown antitumor activity in preclinical studies; however, its toxicity to normal cells, especially within the hematopoietic system, limits its clinical applicability [54–56]. Combining CDK inhibitors with other cancer therapies, such as chemotherapy or immune checkpoint inhibitors, may enhance their overall therapeutic efficacy [56].

In this study, IPA revealed that the PI3K/AKT signaling pathway plays a significant role in the downstream molecular events following shCACYBP treatment in NCI-H1299 cells. The PI3K/AKT pathway is a critical intracellular signaling cascade, and its dysregulation has been linked to cell proliferation, invasion, autophagy, and metastasis in LUAD [57]. Notably, activation of this pathway has been identified as an early pathogenic event in the tumorigenesis and progression of NSCLC [58, 59]. Rescue experiments demonstrated that treatment with the PI3K inhibitor LY294002 partially reversed the effects of CDK1 on promoting proliferation and inhibiting apoptosis in A549 and NCI-H1299 cells. These findings support our hypothesis that CDK1 may contribute to the tumorigenic potential of LUAD cells, at least in part, through the PI3K/AKT pathway, and suggest that this pathway could be involved in CACYBP-mediated LUAD progression.

Our work has several limitations. First, although we demonstrated that CDK1 is involved in the process by which CACYBP knockdown inhibits the malignant progression of LUAD, the specific binding site and exact binding mechanism between CACYBP and CDK1 remain unclear. Second, our findings regarding the PI3K/AKT signaling pathway are preliminary, and the detailed mechanism through which CACYBP regulates this pathway via CDK1 requires further investigation. These issues are the focus of ongoing studies (Figure S3 and S4). Despite these limitations, our study offers novel insights into the role of CACYBP and suggests that targeting CACYBP with RNAi may represent a promising strategy for managing LUAD. Future translational efforts should prioritize the development of CACYBP-targeted therapies and explore their integration with existing CDK1 or PI3K/AKT inhibitors to improve outcomes for patients with advanced LUAD.

## Conclusion

Our findings revealed that elevated CACYBP expression in LUAD is associated with advanced neoplasia and poor prognosis. Knockdown of CACYBP significantly inhibited the growth of LUAD cells. Notably, CACYBP may play a carcinogenic role in LUAD, at least in part by targeting CDK1 to activate the PI3K/AKT signaling pathway. Therefore, CACYBP could serve as a potential prognostic marker or therapeutic target in LUAD.

## Supplemental data

Supplemental data are available at the following link: https://www.bjbms.org/ojs/index.php/bjbms/article/view/11849/3788.

## Data Availability

The datasets used and/or analyzed during the current study are available from the corresponding author on reasonable request.
